# Increased Longevity and Dormancy of Soil-Buried Seeds from Advanced Crop–Wild Rice Hybrids Overexpressing the *EPSPS* Transgene

**DOI:** 10.3390/biology10060562

**Published:** 2021-06-20

**Authors:** Xiao-Qi Jiang, Xiao Yang, Bao-Rong Lu

**Affiliations:** 1 Ministry of Education Key Laboratory for Biodiversity and Ecological Engineering, School of Life Sciences, Fudan University, Songhu Road 2005, Shanghai 200438, China; 15110700007@fudan.edu.cn; 2 Institute of Plant Physiology and Ecology, Shanghai Institutes for Biological Sciences, Chinese Academy of Sciences, Fenglin Road 300, Shanghai 200032, China; yangxiao@sibs.ac.cn

**Keywords:** *Oryza rufipogon*, transgene flow, fitness benefit, soil seed bank, auxin, environmental biosafety assessment

## Abstract

**Simple Summary:**

Estimating the survival and reproductive ability caused by a transgene moved from a genetically engineered (GE) crop to its wild relative populations through gene flow plays an important role in assessing the potential environmental risks of the GE crop. Such estimation has essentially focused on the survival and reproduction-related characteristics above the ground, but with little attention to the GE seeds shattered in the soil seed banks. We demonstrated that the herbicide-resistant transgene overexpressing the rice endogenous EPSP enzyme increased the survival and longevity of the GE crop–wild (*Oryza rufipogon*) hybrid seeds in soil seed banks. In addition, enhanced survival and longevity of the GE hybrid seeds are likely associated with increases in seed dormancy and a growth hormone (auxin) via overexpressing the *EPSPS* transgene. Therefore, the *EPSPS* transgene can persist in the soil seed banks and spread in the environment, causing unwanted environmental impacts.

**Abstract:**

Estimating the fitness effect conferred by a transgene introgressed into populations of wild relative species from a genetically engineered (GE) crop plays an important role in assessing the potential environmental risks caused by transgene flow. Such estimation has essentially focused on the survival and fecundity-related characteristics measured above the ground, but with little attention to the fate of GE seeds shattered in the soil seed banks after maturation. To explore the survival and longevity of GE seeds in soil, we examined the germination behaviors of crop–wild hybrid seeds (F_4_–F_6_) from the lineages of a GE herbicide-tolerant rice (*Oryza* *sativa*) line that contains an endogenous *EPSPS* transgene hybridized with two wild *O. rufipogon* populations after the seeds were buried in soil. The results showed significantly increased germination of the GE crop–wild hybrid seeds after soil burial, compared with that of the non-GE hybrid seeds. Additionally, the proportion of dormant seeds and the content of the growth hormone auxin (indole-3-acetic acid, IAA) in the GE crop–wild hybrid seeds significantly increased. Evidently, the *EPSPS* transgene enhances the survival and longevity of GE crop–wild rice seeds in the soil seed banks. The enhanced survival and longevity of the GE hybrid seeds is likely associated with the increases in seed dormancy and auxin (IAA) by overexpressing the rice endogenous *EPSPS* transgene. Thus, the fate of GE seeds in the soil seed banks should be earnestly considered when assessing the environmental risks caused by transgene flow.

## 1. Introduction

Many genetically engineered (GE) crops with increased insect resistances, herbicide tolerances, and improved qualities are released to the environment for commercial production. To date, GE crops are cultivated in 29 countries with a total cultivation area of >190 million hectares [1]. However, the extensive environmental release of GE crops has aroused tremendous concerns over the environmental biosafety issues, including transgene flow from a GE crop to the populations of its wild relative species [2,3,4,5,6,7,8]. Studies have shown that the major crop species, including wheat, rice, maize, soybean, and oil rapes, can hybridize spontaneously with their wild relatives occurring in the vicinity [3,9,10,11,12,13]. Pollen-mediated crop-to-wild transgene flow or hybridization may result in unwanted environmental and ecological consequences if the transgene acquired by the wild relative populations can confer considerable fitness benefits or costs [4,6,8,14,15,16]. Therefore, assessing the potential environmental impact caused by transgene flow becomes a basic requirement as a regulatory procedure before any GE crop is cultivated for commercial production.

Given that the frequency of transgene flow to a particular wild relative species is properly determined, the next key procedure is to estimate the survival and fecundity of the wild relative plants or populations that have acquired a transgene, namely, estimate the fitness effect conferred by the transgene [6,7,8,17]. If the transgenic characteristics (e.g., herbicide tolerance) enhance the fitness of the wild relative plants or populations carrying the transgene under specific selection pressure, the transgene may increase the likelihood of its persistence and spread in the wild relative populations, causing potential environmental impacts [15,18,19,20,21]. On the other hand, if a transgene brings about negative or harmful effects under natural conditions, it may also cause environmental impacts because the transgene reduces the fitness of the wild relative populations that have acquired the transgene through pollen-mediated gene flow [22,23].

Usually, transgenic fitness is estimated involving hybrid descendants derived from artificial crosses between a GE crop and wild relative species, simulating transgene flow from a GE crop to wild populations [14,15,19,21,24]. Undoubtedly, results generated from these studies have contributed significantly to the assessment of the potential environmental impact caused by transgene flow. Nearly all the studies focused only on the comparison of survival and fecundity-related characteristics between GE ad non-GE crop–wild hybrid lineages, particularly on the number of seeds produced by plants containing a transgene [14,15,19,21]. Such comparison is based on the general assumption that the number of seeds produced by a plant is the key to determining the population size of the crop–wild hybrids. In other words, transgenic plants producing more seeds will establish larger populations than their non-transgenic counterparts. However, very few studies have paid attention to the survival and longevity of the seeds that shattered in the soil seed banks after maturation, although the survived seeds in the soil also play an essential role in sustaining and expanding a plant population for subsequent generations.

**Figure 1:**
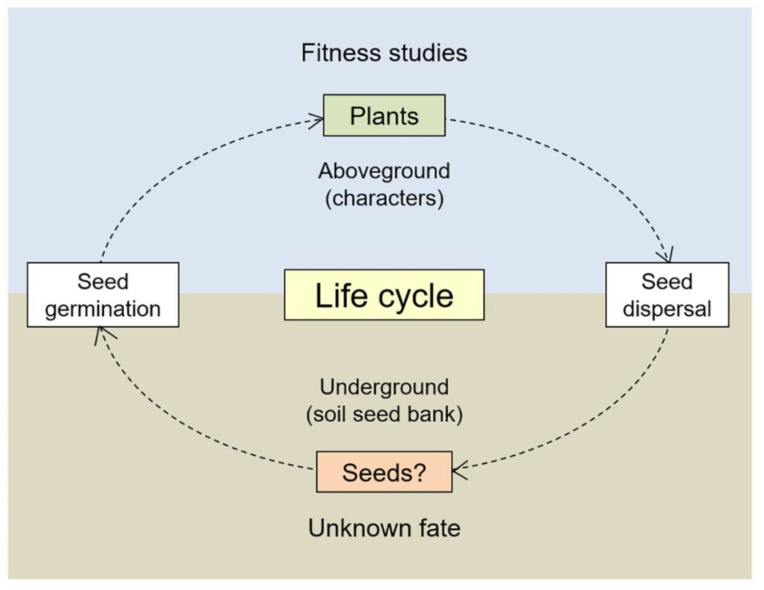
A schematic illustration showing a seed-to-seed life cycle of higher plants. A germinated seed develops into a mature plant that produces next-generation seeds dispersed in the soil seed banks. Studies for transgenic fitness focus essentially on the aboveground characteristics. However, the potential environmental impact of transgenic seeds remaining in the soil seed banks, which determines the overall fitness of plant populations, is almost unknown.

Seeds that maintain in the soil seed banks represent an essential stage in the seed-to-seed life cycle of a higher plant (Figure 1). The dynamics of seed populations in the soil seed banks determine the number of seedlings and plants for the next-generation populations, which constitutes a critical part of the overall fitness of a plant population in the complex environment [2]. Therefore, understanding the survival and longevity of seeds in the soil seed banks provides an important basis for the correct assessment of the environmental impact caused by transgene flow to populations of wild relative species. The survival and longevity of seeds in the soil seed banks can be influenced by genetic factors, such as the seed-coat colors and seed dormancy [25], in addition to environmental factors. Seed dormancy, preventing seeds from random germination, allows seeds of a plant to survive and persist in the soil seed banks [26]. In fact, seeds that have strong dormancy were found to have better survival and longevity in the soil seed banks [27,28]. Consequently, plant populations with better seed survival and longevity have better opportunities to sustain and expand in their natural habitats [27,29].

Previous studies reported significantly enhanced fecundity in GE hybrid lineages derived from crosses between a GE rice (*Oryza sativa*) line and wild (*O. rufipogon*) or weedy rice (*O. sativa* f. *spontanea*) populations [15,21]. The rice line that was developed to tolerate the glyphosate herbicide contained a rice endogenous *EPSPS* (5-enolpyruvoylshikimate-3-phosphate synthase) transgene overexpressing the EPSP enzyme [30]. Further studies indicated that overexpressing the *EPSPS* transgene considerably increased seed production of GE hybrids under the glyphosate free conditions, in addition to increases in the content of chlorophyll, lignin, auxin (indole-3-acetic acid, IAA), and tryptophan [15,31,32,33,34]. These results indicate that the transgene overexpressing *EPSPS* may cause considerable environmental impacts if it is incorporated into populations of crop wild relative species through pollen-mediated gene flow.

Studies on *Arabidopsis* and rice containing the transgene overexpressing *EPSPS* from different sources demonstrated substantial transgenic benefits, with only a slight position effect of gene insertion between different transgenic events [31,32]. However, no fitness effect was reported by the hygromycin resistant gene used as a selecting marker [31]. These results suggest potential environmental impacts of the *EPSPS* transgene if it is transferred into the populations of the crop’s wild/weedy relatives through gene flow. However, whether the transgenic seeds in soil seed banks contribute to increased fitness is still unknown, which will considerably affect the accurate assessment of the environmental impacts caused by transgene flow.

To further understand the environmental impact from the shattered GE seeds containing the *EPSPS* transgene in the soil seed banks, we produced F_3_–F_5_ crop–wild hybrid lineages derived from an *EPSPS* transgenic rice line (EP3) crossed with two wild rice populations [15], following the widely adopted method to study the fitness effect caused by a transgene [14,15,21,35,36]. We estimated the survival and longevity of the GE and non-GE hybrid seeds (F_4_–F_6_) derived from the hybrid lineages by determining their germination ratios after soil burial treatments. We also examined dormancy and the hormone contents in the seeds. The primary objectives of this study were to answer the following questions. (i) Does the transgene overexpressing *EPSPS* considerably increase the survival and longevity of crop–wild rice hybrid seeds shattered in soil? (ii) Can this *EPSPS* transgene change dormancy of the hybrid seeds? (iii) Does the content of plant hormones vary in the crop–wild hybrid seeds with the change of seed dormancy? Answers to these questions can help us to correctly estimate the possible persistence and spread of *EPSPS* transgene in wild rice populations and to assess the ecological and environmental impacts caused by transgene flow.

## 2. Materials and Methods

### 2.1. Plant Materials

An herbicide-resistant GE rice line (EP3, pollen donor) and two wild rice populations (w1 and w2, pollen recipient) were used to produce crop–wild rice hybrid descendants (F_3_–F_5_). The GE EP3 rice (*Oryza sativa*) line contained the endogenous transgene that overexpressed the 5-enolpyruvoylshikimate-3-phosphate synthase (EPSPS) and was developed from a rice variety Minghui-86 by *Agrobacterium*-mediated transformation to tolerate the glyphosate herbicide [30]. The two wild rice (*Oryza rufipogon*) populations were collected from Dongxiang in the Jiangxi province (w1) and Suixi in the Guangdong province (w2) in China, respectively. Approximately 20 plants from each of the two wild rice populations (as pollen recipients) were included to produce the F_1_ hybrid progenies. Five F_1_ hybrid plants from each of the hybrid combinations were randomly selected to form a mixed balk population with ~100 plants to develop the successive hybrid progenies.

**Figure 2:**
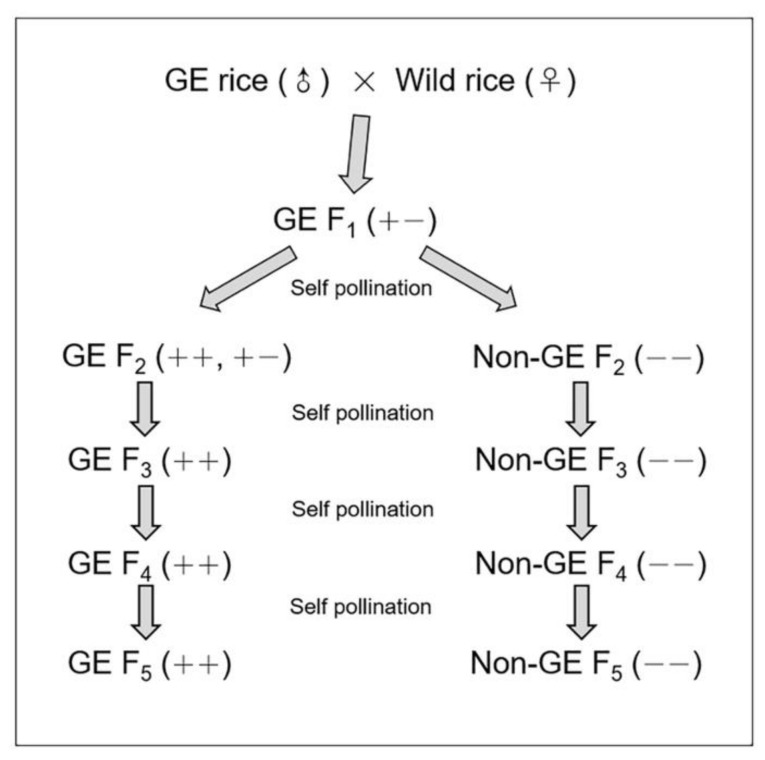
An illustration showing the production and pedigrees of GE rice (EP3) × wild rice (w1 or w2) hybrids and the derived F_2_–F_5_ hybrid lineages through successive self-pollination. + + and + − indicate GE homozygous and heterozygous hybrids/lineages, respectively; − − indicates non-GE homozygous hybrids/lineages.

The homozygous hybrid lineages (Figure 2) with (GE) or without (non-GE) the *EPSPS* transgene were obtained from self-bred hybrid descendant plants following the method of Yang et al. [15]. Approximately 100 GE or non-GE homozygous hybrid plants from hybrid progenies of each generation were randomly selected to form GE and non-GE hybrid lineages by identifying the presence or absence of the *EPSPS* transgene [15]. Because of the relatively large hybrid population size and randomly sampling procedure for GE and non-GE plants, the resulted GE and non-GE hybrid lineages of different generations should have statistically the same genetic background following Mendel’s law of random segregation. Therefore, the detected differences between the GE and non-GE hybrid lineages represented those from the transgene [14,15,21,35,36]. In this experiment, the F_4_–F_6_ hybrid seeds produced from F_3_–F_5_ plants of GE and non-GE hybrid lineages (Figure 2) were used to determine the effect of the *EPSPS* transgene on seed germination, dormancy, and contents of plant hormones.

### 2.2. Seed Dormancy Breaking

Seed dormancy-breaking treatment was conducted to the crop–wild hybrid seeds for removing seeds dormancy before soil burial treatment or germination examination, using GA_3_ solution (gibberellic acid 3 from BBI Life Sciences Corporation, Shanghai, China). In the treatment, hybrid seeds were soaked in 72.2 μM GA_3_ (0.25 g/L) solution for 24 h to break seed dormancy and cleaned with distilled water after the dormancy-breaking treatment, and then soaked in distilled water for 8 h to remove the residual gibberellin solution.

### 2.3. Soil Burial Treatment

Soil burial treatments, which simulated the shattered seeds in the soil seed banks, were conducted in a dark growth chamber with the controlled temperature at ~10 °C on GE and non-GE F_4_–F_6_ crop–wild hybrid seeds. Two experiments were conducted: without or with a dormancy-breaking treatment before seeds were buried in soils. Five treatments were included in the soil-burial experiments: 0, 25, 50, 75, and 100 days to determine the fitness effect of the *EPSPS* transgene in the soil seed banks by comparison between GE and non-GE hybrid lineages in a particular treatment. For each treatment, ~240 seeds sampled separately from GE and non-GE crop–wild hybrid lineages were included for each treatment and divided into six replicates. Seeds of each replicate were included in a fine-mesh nylon bag and then buried at ~5 cm depth in the soil, ensuring the close contact of soil and seeds. The control seeds (0 days) were kept at room temperature for ~24 h after being taken out of the 4 °C refrigerator before seed germination.

### 2.4. Examination of Seed Germination and Dormancy

Seed germination was examined after the buried seeds were washed by distilled water and sterilized with 75% ethanol. The clean seeds were germinated on moist filter paper at 28 °C in a dark growth chamber. The germination ratios (the number of germinated seeds divided by all seeds exposed to germination) were determined after the seeds were exposed to the germination condition for 10 days. Here, the seed germination ratio was used to estimate the survival ratio of seeds at a point after an experimental treatment, whereas the seed survival ratio was used to estimate the seed longevity after a series of experimental treatments for a certain duration.

Ungerminated seeds were exposed to the 2,3,5-triphenyltetrazolium chloride (TTC) staining test following the Lakon description [37] to determine seed dormancy. The ungerminated seeds were cut longitudinally to expose their embryos, and then the entire seeds were stained in the 0.1% TTC solution (from BBI Life Sciences Corporation, Shanghai, China) for 2 h at ~25 °C in darkness. The seeds that showed stainable bright red embryos were determined as viable (dormant) seeds, whereas those that showed white embryos were considered dead seeds. The dormancy ratio was determined by the number of stained seeds divided by all seeds exposed to germination after the TTC staining test.

### 2.5. Examination of Plant Hormones in Seeds

The contents of the plant hormones were examined in GE and non-GE F_4_–F_6_ hybrid seeds based on the high-performance liquid chromatography tandem mass spectrometry (HPLC-MS) method. The contents of six germination or dormancy-related plant hormones, including ABA (abscisic acid), *cis*-OPDA (*cis*-(+)-12-oxo-phytodienoic acid), GA_3_, IAA (indole-3-acetic acid), JA (jasmonic acid), and SA (salicylic acid), were examined in both dry and imbibed seeds. For each treatment, 150 hybrid seeds were randomly selected from GE and non-GE crop–wild hybrid lineages and divided into three replicated samples on average for each treatment. The untreated dry seeds were directly taken out from 4 °C refrigerators. The imbibed seeds were placed on moist filer papers for 8 h at ~10 °C to avoid random germination.

For hormone content examination, ~80 mg frozen-powdered seeds of each sample were mixed with 50 μL of internal standard solution and 1 mL of acetonitrile water solution with 1% of formic acid. The mixture was extracted for 12 h at ~4 °C. Then, the mixture was centrifuged at 14,000× *g* for 10 min. Approximately 800 μL supernatant was taken, nitrogen-dried, and dissolved in 100 μL of 1% acetonitrile solution. The solution was centrifuged at 14,000× *g* for 10 min, and the supernatant was taken as extracted test samples.

The test samples were separated by liquid chromatography using the Waters I-Class Ultra High-Performance Liquid Chromatography System (Waters Inc., Milford, MA, USA). In total, 20 μL of the test sample solution was separated at a flow ratio of 400 μL/min. In the liquid chromatography separation, we used 0.05% formic acid water solution as eluent solvent A and 0.05% formic acid acetonitrile solution as eluent solvent B for the mobile phase. The concentration of solvent B in eluent began with 2%, raised linearly to 98% at 10 min, then reduced linearly to 2% at 11.1 min, and kept at 2% between 11.1 and 13 min. The mass spectrometric analysis was conducted on test samples and standard samples (supplied by OIChemIm from Shanghai, China) by the 5500 QTRAP Mass Spectrometer (AB SCIEX Inc., Framingham, MA, USA) with the following electrospray ionization (ESI) parameters: source temperature 500 °C, ion Source Gas1 (Gas1): 45, ion Source Gas2 (Gas2): 45, curtain gas (CUR): 30, ion sapary voltage floating (ISV)-4500 V. The absolute quantity of hormones in the samples was obtained using the software MultiQuant (AB SCIEX Inc., Framingham, MA, USA).

### 2.6. Statistical Analysis

The independent-sample Student’s *t*-test was conducted to compare differences in the survival and fecundity related characteristics, including seed germination ratios, seed dormancy ratios, and hormone contents in seeds between GE and non-GE hybrid lineages. In addition, the same *t*-test was conducted to compare differences in germination ratios between seeds with or without dormancy-breaking treatments. All statistical analyses were performed using the software SPSS ver. 22.0 (IBM Inc., New York, NY, USA).

## 3. Results

### 3.1. Survival and Longevity of GE and Non-GE Crop–Wild Hybrid Seeds After Soil Burial Treatments

**Figure 3:**
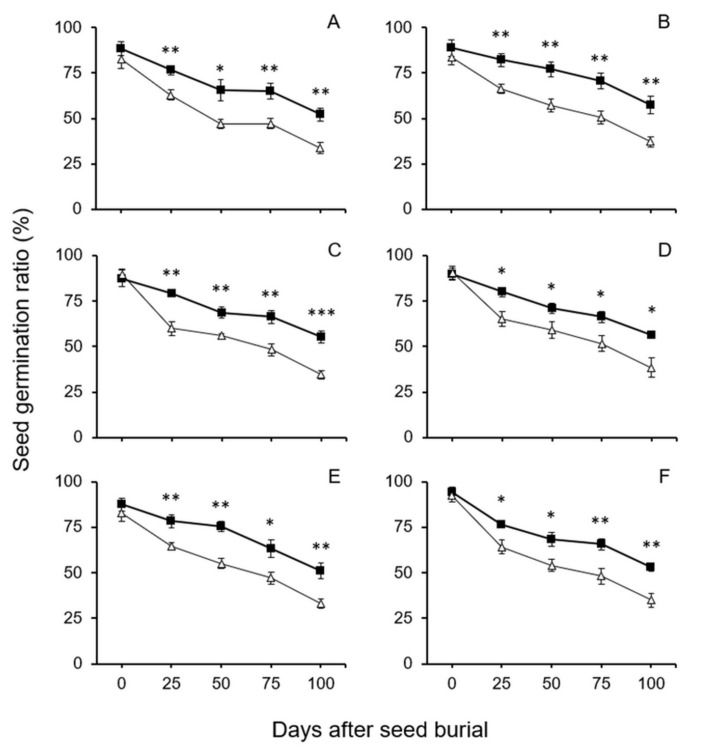
Average germination ratios of GE and non-GE seeds (F_4_–F_6_) from the transgenic crop (EP3)–wild rice (w1 or w2) hybrid lineages after soil burial treatments at 0, 25, 50, 75 and 100 days. No dormancy-breaking treatment was conducted before seed burial. (**A**,**B**) F_4_ of EP3–w1 and EP3–w2; (**C**,**D**) F_5_ of EP3–w1 and EP3–w2; (**E**,**F**) F_6_ of EP3–w1 and EP3–w2. Comparisons were made between GE (solid squares) and non-GE (empty triangles) seed germination based on the independent-sample Student’s *t*-test. Bars represent standard error (*n* = 6 replicates). * *p* < 0.05, ** *p* < 0.01.

In the seed longevity experiment with crop–wild hybrid seeds with dormancy, all seeds from refrigerator storage were directly buried in the soil without dormancy-breaking. After being treated in the GA_3_ solution to break seed dormancy, the soil-burial-treated seeds (25~100 days) and controls (0-day treatment) were germinated for examining the germination ratios. Thus, the measured seed germination ratios represented the survival and longevity of all hybrid seeds regardless of seeds with or without dormancy. The results of the experiment showed no significant differences in germination ratios between the seeds from the GE and non-GE lineages derived from crop–wild hybrids before the soil-burial treatment (Figure 3, 0-day treatment). In addition, the germination ratios of the crop–wild hybrid seeds before soil-burial treatment were high, ranging from 83% to 93%. These results indicated that overexpressing the *EPSPS* transgene did not have a significant effect (*p* > 0.05, independent Student’s *t*-test) on the longevity of the GE crop–wild hybrid seeds under the condition in normal refrigerator storage conditions.

In contrast, after these seeds were buried in soil for 25, 50, 75, and 100 days, all the GE crop–wild hybrid seeds from different generations (F_4_–F_6_) showed significantly higher germination ratios (*p* < 0.05, independent Student’s *t*-test) than their non-GE counterparts after dormancy-breaking (Figure 3). These results suggested that overexpressing the *EPSPS* transgene significantly increased the survival and longevity of the GE crop–wild hybrid seeds after the seeds shattered and buried in the soil seed banks. Interestingly, the seed germination ratios of all the buried seeds gradually decreased from ~71% (25-day burial treatments, Figure 3) to ~45% (100-day burial treatments, Figure 3). These results indicated an obvious trend that the survival ratios of the hybrid seeds gradually reduced with the increased time when the crop–wild hybrid seeds shattered and buried in the soil seed banks.

**Figure 4:**
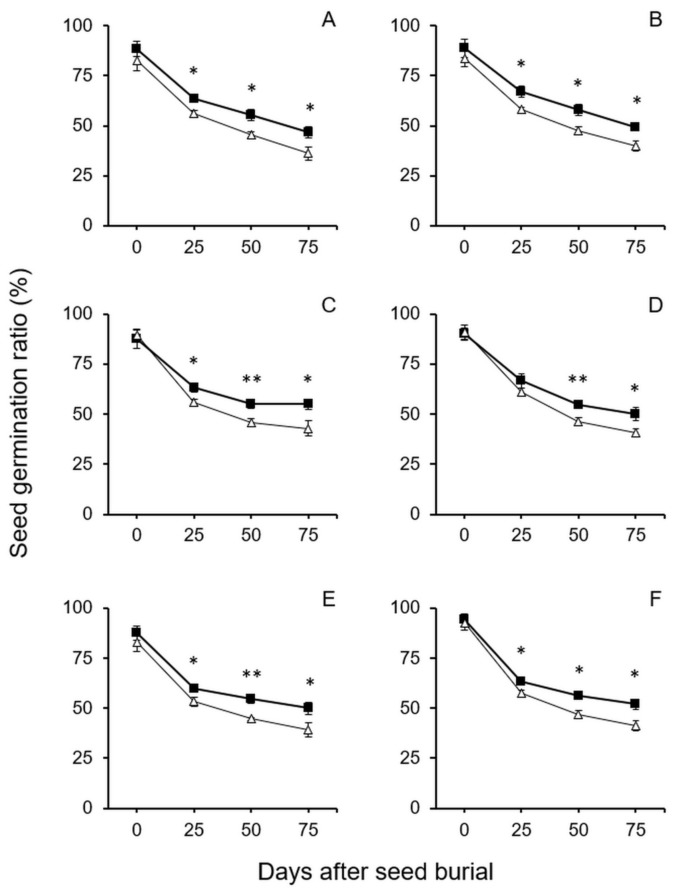
Average germination ratios of GE and non-GE seeds (F_4_–F_6_) from the transgenic crop (EP3)–wild rice (w1 or w2) hybrid lineages after soil burial treatments at 0, 25, 50, and 75 days. The dormancy-breaking treatment was conducted before seed burial. (**A**,**B**) F_4_ of EP3–w1 and EP3–w2; (**C**,**D**) F_5_ of EP3–w1 and EP3–w2; (**E**,**F**) F_6_ of EP3–w1 and EP3–w2. Comparisons were made between GE (solid squares) and non-GE (empty triangles) seed germination based on the independent-sample Student’s *t*-test. Bars represent standard error (*n* = 6 replicates). * *p* < 0.05, ** *p* < 0.01.

In the seed longevity experiment including crop–wild hybrid seeds without dormancy, all the refrigerator-stored seeds were obtained and then treated by GA_3_ solution to artificially break seed dormancy before the seeds were buried in soil. Similar to the results of soil-burial-treated seeds with dormancy, this experiment also showed that nearly all the F_4_–F_6_ GE crop–wild hybrid seeds (excepting for F_5_ seeds from the EP3–w2 hybrid lineage in the 25-day soil burial treatment) without dormancy had significantly higher seed germination ratios (*p* < 0.05, independent Student’s *t*-test) than their non-GE counterparts after buried in soil for 25, 50, and 75 days (Figure 4). Likewise, there was also an obvious trend that the survival ratios of the dormancy-broken hybrid seeds gradually reduced with the increases in the length of the seeds treated in soil burial.

Altogether, these results suggested that overexpressing the *EPSPS* transgene would consistently increase the survival ratios and longevity of the GE crop–wild hybrid seeds after they shattered and were buried in soil for a certain period of time, regardless of the seeds with or without primary dormancy in the treatment.

### 3.2. Dormancy of GE and Non-GE Crop–Wild Hybrid Seeds

**Figure 5:**
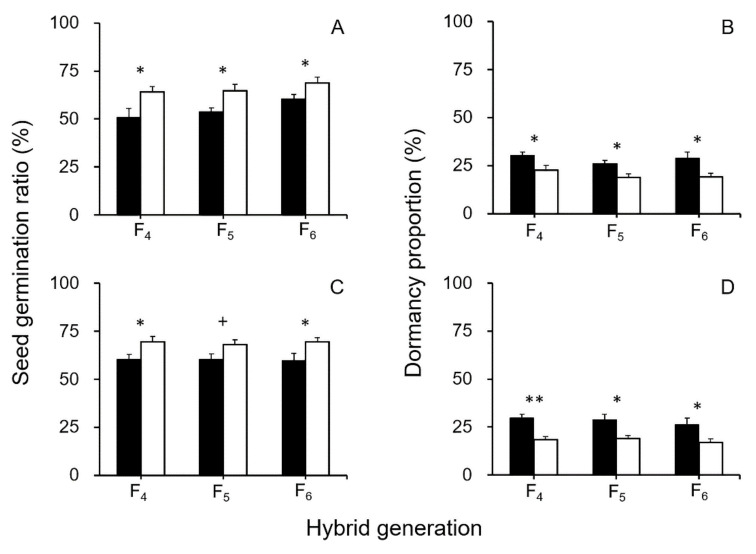
Average seed germination and dormancy ratios of the crop (EP3)–wild rice (w1 or w2) hybrid seeds (F_4_–F_6_). All seeds were not treated both with dormancy breaking and soil burial. (**A**,**C**) seed germination ratios of EP3–w1 and EP3–w2; (**B**,**D**) seed dormancy ratios of EP3–w1 and EP3–w2. Comparisons were made between GE (black columns) and non-GE (white columns) seeds based on the independent- sample Student’s *t*-test. Bars represent standard error (*n* = 6 replicates). + *p* < 0.1, * *p* < 0.05, ** *p* < 0.01.

The observed germination ratios of GE and non-GE crop–wild hybrid seeds were relatively high (83–93%) for the control (0-day treatment, in Figure 3 and Figure 4) after the dormancy-breaking treatment. However, the germination ratios of the seeds without the dormancy-breaking treatment were relatively low (51–70%) for the control, in which seeds with dormancy were excluded (0-day treatment, Figure 5A,C). Differences in germination ratios between the two sets of results indicated that the presence of primary dormancy in the GE and non-GE crop–wild hybrid seeds influenced their germination ratios. Noticeably, the GE crop–wild hybrid seeds had significantly lower germination ratios (*p* < 0.10, independent-sample Student’s *t*-test) than the non-GE crop–wild hybrid seeds (Figure 5A,C). These results suggested considerably higher seed dormancy ratios in GE crop–wild hybrid seeds than that of non-GE hybrid seeds.

The above observation was supported by further TTC staining test of the ungerminated seeds without the soil-burial treatment, where ~28% stainable seeds were detected in GE crop–wild hybrid lineages and ~19% stainable seeds were detected in non-GE hybrid lineages (Figure 5B,D). The results confirmed the higher seed dormancy ratios in the GE hybrid lineages than in the non-GE hybrid lineages of all generations (F_4_–F_6_), with significant differences (*p* < 0.05, independent-sample Student’s *t*-test). In addition, results from the TTC staining test of the ungerminated seeds treated by 25-day soil burial showed significantly more stainable seeds, namely significantly higher dormancy ratios (*p* < 0.05, independent-sample Student’s *t*-test, Table 1) in the GE hybrid seeds than in their non-GE counterparts. This finding showed a similar tread to that of hybrid seeds without soil-burial treatment. Altogether, these results indicated that overexpression of the *EPSPS* transgene increased the proportions of dormant seeds from all the GE crop–wild hybrid lineages, regardless of whether or not the seeds were treated by soil burial (Figure 5B,D).

**Figure 6:**
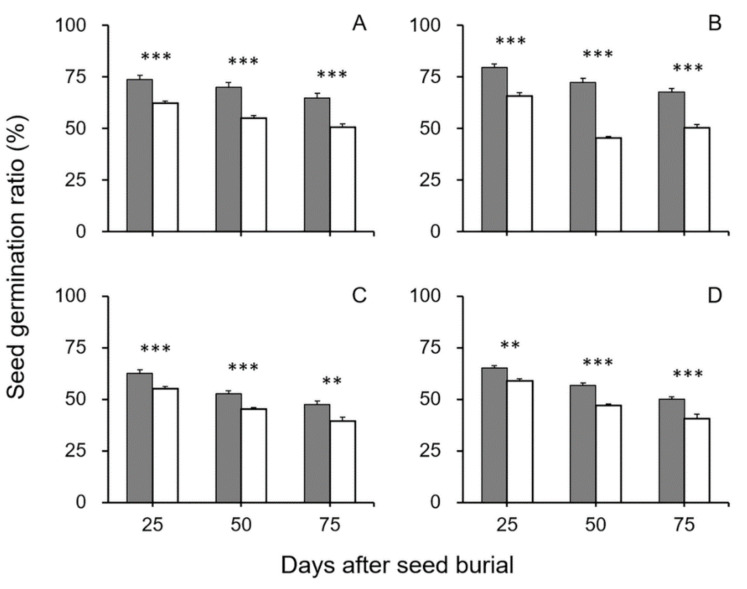
Average germination ratios of the crop (EP3)–wild rice (w1 or w2) hybrid seeds after soil burial treatments at 25, 50, and 75 days. (**A**,**B**) GE seeds of EP3–w1 and EP3–w2; (**C**,**D**) non-GE seeds of EP3–w1 and EP3–w2. Comparisons were made between seeds without (grey columns) and with (white columns) dormancy breaking treatment based on the independent-sample Student’s *t*-test. Bars represent standard error (*n* = 18 replicates). ** *p* < 0.01, *** *p* < 0.001.

In addition, our results further demonstrated a significantly greater level of germination (*p* < 0.01, independent-sample Student’s *t*-test) for the crop–wild hybrid seeds without the dormancy breaking treatment than those with the dormancy breaking treatment, regardless of GE or non-GE hybrid lineages (Figure 6), after the seeds were buried in soil for 25, 50, and 75 days. These results suggested that *EPSPS* transgene-induced seed dormancy alone had a significant effect (*p* < 0.01, independent-sample Student’s *t*-test) on the survival and longevity of GE hybrid seeds shattered in soil. In other words, the GE crop–wild hybrid seeds of different generations (F_4_–F_6_) with stronger dormancy had a greater level of survival and longevity than those with less dormancy after the seeds shattered in the soil.

Furthermore, we also found that all the crop–wild hybrid seeds, of which dormancy was broken by the GA_3_ treatment before soil burial, did not recuperate their dormancy after the seeds were buried in the soil. This result was obtained based on the TTC staining test, where all ungerminated seeds were proven to be unstainable.

### 3.3. Changes of Plant Hormones in GE and Non-GE Crop–Wild Hybrid Seeds

**Figure 7:**
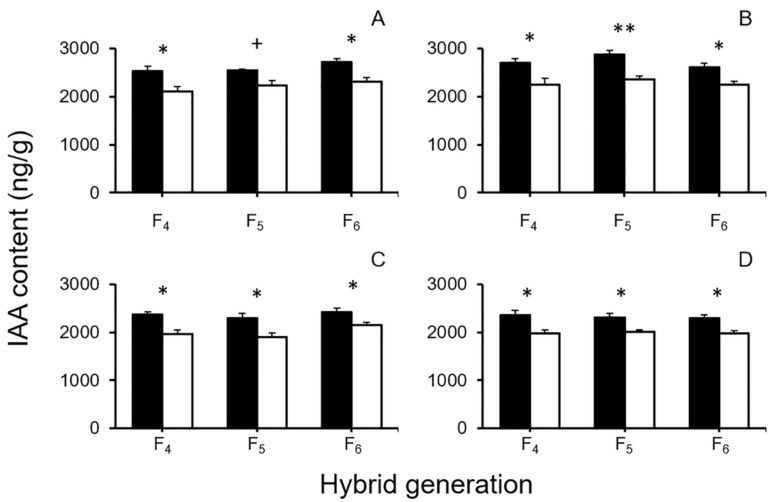
Average IAA contents of GE and non-GE crop (EP3)–wild rice (w1 or w2) hybrid seeds (F_4_–F_6_). (**A**,**B**) dry seeds of EP3–w1 and EP3–w2; (**C**,**D**) imbibed seeds of EP3–w1 and EP3–w2. Comparisons were made between GE (black columns) and non-GE (white columns) seeds based on the independent-sample Student’s *t*-test. Bars represent standard error (*n* = 3 replicates). + *p* < 0.1, * *p* < 0.05, ** *p* < 0.01.

The contents of six plant hormones that were in some way associated with seed activities, including germination and dormancy, such as ABA, *cis*-OPDA, GA_3_, IAA, JA, and SA, were examined in F_4_–F_6_ seeds from the GE and non-GE crop–wild hybrid lineages, respectively. The hormones were examined in seeds under dry (untreated) and imbibed conditions using the HPLC-MS method. Among the six plant hormones, the content of IAA showed significantly higher average values (*p* < 0.05, independent-sample Student’s *t*-test) in all the GE hybrid seeds than that in the non-GE hybrid seeds, under both the dry (Figure 7A,B) and imbibed (Figure 7C,D) conditions, although dry seeds from the EP3–w1 F_5_ hybrid lineage showed a close to significance (*p* < 0.10). These results evidently indicated that overexpressing the *EPSPS* transgene significantly increased the contents of IAA in the GE crop–wild hybrid seeds under both dry and imbibed conditions.

In addition, the contents of the other five plant hormones did not show a consistent pattern of differences (*p* > 0.05, independent-sample Student’s *t*-test) in GE and non-GE crop–wild hybrid seeds under both the dry or imbibed conditions (Table 2 and Table 3). For example, under the dry condition, the GE hybrid seeds from the F_4_ EP3–w1 and F_5_ EP3–w2 hybrid lineages showed a significantly higher level of ABA contents (*p* < 0.05, independent-sample Student’s *t*-test) than their non-GE hybrid seeds, whereas the GE hybrid seeds from the F_4_ EP3–w1 hybrid lineage showed a significantly lower level of JA content (*p* < 0.01, independent-sample Student’s *t*-test) than their non-GE seeds (Table 2). These results suggested that overexpressing the *EPSPS* transgene did not have a consistent effect on the contents of the five examined plant hormones, namely, ABA, *cis*-OPDA, GA_3_, JA, and SA.

## 4. Discussion

### 4.1. EPSPS Transgene Increases the Survival and Longevity of GE Crop–Wild Hybrid Seeds in Soil Seed Banks

In general, our results showed significant increases in germination ratios of the GE crop–wild hybrid seeds (F_4_–F_6_), after these seeds were buried in soil for a different duration of time (25–100 days), compared with those of non-GE hybrid seeds. These results suggest that overexpressing of the *EPSPS* transgene can significantly enhance the survival and longevity of the GE seeds derived from crop–wild rice hybrid descendants, after the seeds shattered in the soil seed banks. Although with a certain degree of variation in hybrid combinations involving the two wild rice parents, this finding addressed our first question regarding overexpression of the *EPSPS* transgene that greatly increased fitness of the crop–wild hybrid seeds in the soil seed banks. In addition, the performance of the GE hybrid seeds with increased soil-stress tolerances conferred by the transgene is consistent, regardless of whether primary seed dormancy was artificially removed or not by the dormancy-breaking treatments. To our knowledge, this is the first study to document the increased survival and longevity of GE rice crop–wild hybrid seeds in the soil seed banks, conferred by a transgene that overexpressing *EPSPS* for glyphosate tolerance [21,30,31,38].

Based on the consistent performance of the crop–wild hybrid seeds from three successive generations, we expect that the increased fitness benefit of GE crop–wild hybrid rice seeds conferred by the *EPSPS* transgene may continue to increase the adaptation and competition potentials of the GE crop–wild rice hybrid seeds in the soil seed banks. Consequently, the increased GE seed populations in the soil seed banks will not only promote the long-term persistence and spread of the *EPSPS* transgene but also sustain and support a larger size of GE crop–wild populations in natural habitats where the native wild rice populations inhabit. Given that the reported crop–weedy/wild hybrids containing the transgene overexpressing *EPSPS* already obtained enhanced fecundity, even under the glyphosate free condition [15,21], the increased survival and longevity of GE hybrid seeds containing the same transgene will further complicate the unexpected environmental impact after the seeds shattered in the soil seed banks. Many previous studies of the fitness benefit conferred by a transgene focus essentially on estimating the survival and fecundity of hybrids derived from crosses between a GE crop and its wild relatives [4,8,14,15,19,21,24]. Results obtained from this study demonstrate the critical role of transgenic seeds in the soil seed banks, as an important phase of the entire plant life cycle, in sustaining transgenic crop–wild hybrid populations.

Therefore, we predict that the GE crop–wild hybrids that contain the transgene overexpressing *EPSPS*, probably also other transgenes with natural selective advantages, will not only produce more seeds than their non-GE counterparts but also produce seeds that will persist longer in the soil seed banks, promoting expansion of the GE hybrid populations generated from these seeds. Thus, when assessing the environmental consequences caused by transgene flow to wild and weedy populations, particularly by such performance-enhancing transgenes as *EPSPS* with the increased fitness benefit, the role of the transgenic seeds in the soil seed banks should be considered as an important procedure in the risk assessment.

### 4.2. Overexpressing EPSPS Transgene Increases Dormancy in GE Crop–Wild Hybrid Seeds

Our results from seed dormancy examination showed a significantly increased proportion of dormant seeds derived from the GE crop–wild hybrid linages overexpressing the *EPSPS* transgene, regardless of whether the seeds were treated by soil burial or not. The increases in seed dormancy conferred by the *EPSPS* transgene exhibited a consistent trend in different hybrid generations (F_4_–F_6_). This result addressed our second question regarding the influences of the *EPSPS* transgene on dormancy of the GE crop–wild hybrid seeds, although our knowledge is still limited to confidently explain the underlying mechanisms connecting seed dormancy with the overexpressing *EPSPS* transgene in rice. However, we can explain the linkage between increased seed dormancy and overexpression of the *EPSPS* transgene well through increased tryptophan as reported by Wang et al. and Achary et al. [21,32] and increased IAA by Fang et al. [31], both of which are supposedly responsible for seed dormancy [21,31,32,39]. Our finding perfectly agrees with the previous conclusions that overexpression of the endogenous *EPSPS* gene from cultivated rice enhances the performance of many fitness-related phenotypic characteristics of transgenic plants [15,21] and physiological and metabolic processes, including tryptophan, chlorophyll, and lignin syntheses [21,31,33,34]. To our knowledge, this is also the first report about the increased dormancy in GE crop–wild hybrid seeds conferred by a transgene that overexpresses *EPSPS* used primarily for glyphosate resistance.

Seed dormancy is an important mechanism regulating seed germination at the proper time and space for higher plants [40,41], which prevents seeds from random germination under adverse environmental conditions, such as the cold winters and drought seasons. It is generally recognized that seed dormancy is closely associated with seed longevity, particularly in the soil seed banks, for the higher plants. For example, a few studies already reported that the losses of seed dormancy in some *Arabidopsis* mutants were closely associated with the reduction of the seed longevity [42,43]. In addition, a study of weedy rice (*Oryza sativa* f. *spontanea*) also showed that seeds with stronger dormancy resulted in better survival and greater seed longevity in the soil seed banks [28]. Therefore, we conclude based on our results that the enhanced survival and longevity of the crop–wild GE hybrid seeds in the soil burial treatments may partially be due to the increases in seed dormancy caused by overexpressing the *EPSPS* transgene.

In fact, further results based on the comparison between germination ratios of the crop–wild hybrid seeds with or without the dormancy-breaking treatments before the soil burial suggested that seeds with dormancy showed a higher level of survival ratios and longevity than those without dormancy. This finding means that the proportion of the crop–wild hybrid seeds with better survival in the soil seed banks was likely caused by their increased seed dormancy. Therefore, we consider that increased seed dormancy acts as an important mechanism that influenced the survival and longevity of the GE crop–wild hybrid seeds in the soil seed banks. These results support the above prediction that the increased seed survival and longevity in the soil seed banks is partially due to the enhanced seed dormancy induced by overexpressing of the *EPSPS* transgene. In addition, our results showed that the crop–wild rice hybrid seeds, of which dormancy was removed by the dormancy-breaking treatments, did not regain or induce seed dormancy both in the GE and non-GE hybrid seeds, even after the long-term soil-burial treatments. These results suggest that the soil burial treatments cannot recover dormancy, both in GE and non-GE hybrid seeds, as long as the seeds were artificially treated with the dormancy-breaking reagents, although the mechanism of such phenomenon is yet unclear.

### 4.3. Overexpressing EPSPS Transgene Increases IAA Content in GE Crop–Wild Hybrid Seeds

Our results showed the significantly increased content of auxin (IAA) in the transgenic seeds derived from the F_4_–F_6_ GE crop–wild hybrid lineages, among the six examined plant hormones. The other five hormones (ABA, *cis*-OPDA, GA_3_, JA, and SA) did not show a consistent pattern in terms of their contents. The transgenic fitness effect on the hormone contents, particularly for IAA, was observed both in the inactive dry seeds from a refrigerator (~4 °C) and the activated seeds imbibed under a low temperature (~10 °C) for eight hours. These results suggest that the increased production of the EPSPS enzyme has promoted the biosynthesis and accumulation of IAA in the GE hybrid seeds by overexpressing the *EPSPS* transgene.

IAA is a downstream metabolite synthesized in the shikimate pathway, in which EPSPS encoded by the *EPSPS* gene acts as a key enzyme [44]. An important end-product synthesized through the shikimate pathway is tryptophan, which serves as the essential source material for IAA biosynthesis [45,46]. Therefore, the increased content of IAA in the GE crop–wild hybrid seeds detected in this study can easily be explained by the insertion of the transgene that overexpresses EPSPS in the GE plants. Probably, the overproduction of EPSPS can accelerate the biosynthesis of tryptophan and then IAA in the shikimate pathway of the GE crop–wild hybrid descendants. Previous studies reported that overexpressing the *EPSPS* transgene significantly increased the tryptophan content in the GE-cultivated rice seeds [32] and in the crop–weed hybrid plants derived from an *EPSPS* transgenic rice (*O. sativa*) line (EP3) crossed with four weedy rice (O. *sativa* f. *spontanea*) accessions [21]. In addition, overexpression of the same rice *EPSPS* transgene also significantly increased the IAA content in GE *Arabidopsis* plants [31]. Altogether, these results confirm the important role of the transgene overexpressing *EPSPS* in the biosynthesis of IAA in plants. Likewise, the increases in IAA content detected in the crop–wild hybrid seeds from this study should be closely associated with overexpression of the endogenous *EPSPS* gene from GE-cultivated rice.

Obviously, the increases in IAA content of the GE crop–wild hybrid seeds, which was caused by the overexpressing *EPSPS* transgene, have played an active role in increasing seed dormancy, as observed in this study. This observation is supported by the study in which Liu et al. [47] demonstrated that variation in the IAA contents was associated with seed dormancy in *Arabidopsis*, although IAA influenced seed dormancy of Arabidopsis jointly with ABA. In addition, several studies also reported that exogenous IAA sprayed on the surface of wheat, tobacco and soybean seeds enhanced their seed dormancy [48,49,50]. Together, all the results about the changes in IAA demonstrated that the increased IAA content promoted seed dormancy. Therefore, we believe that the increased IAA content conferred by the transgene overexpressing *EPSPS* in this study enhanced the dormancy of the GE crop–wild rice hybrid seeds, which is partially responsible for the increased survival and longevity of the GE crop–wild hybrid seeds in the soil seed banks. The detailed mechanisms of the *EPSPS* transgene that regulates the biosynthesis of IAA and how IAA influences seed dormancy in rice seeds need further studies.

## 5. Conclusions

In conclusion, results from this study demonstrate that the transgene overexpressing *EPSPS* significantly enhanced the survival and longevity of transgenic seeds from GE rice crop–wild hybrid descendants in different generations (F_4_–F_6_) consistently, after the seeds were buried in soils. In addition, the *EPSPS* transgene significantly increased dormancy of the transgenic hybrid seeds, which is partially responsible for the enhanced survival and longevity of the transgenic seeds in the soil seed banks. Unexpectedly, the transgenic hybrid seeds that had increased dormancy showed considerably increased contents of IAA both under the inactive (dry) and activated (imbibed) conditions among the six examined plant hormones. Therefore, we predict that the enhanced seed survival and longevity conferred by the *EPSPS* transgene will promote the long-term persistence and spread of the transgene in soil seed banks, causing unwanted environmental impacts from crop-to-wild transgene flow. Additionally, more transgenic seeds in the soil seed banks will sustain and even promote much larger wild/weedy rice populations that have already gained enhanced fecundity because of the transgene overexpressing *EPSPS*, promoting more invasive weeds. These findings have important implications in risk assessment of the environmental impacts caused by transgene flow. Given the fact that transgenic crop–wild hybrid seeds may acquire fitness benefit in the soil seed banks, we recommend that estimating the survival and longevity of crop–wild/weedy hybrid seeds in the soil seed banks should be taken into consideration earnestly in the risk assessment of the environmental impacts caused by transgene flow.

## Figures and Tables

**Table 1:** Average dormancy ratios (%) of F_4_–F_6_ seeds derived from hybrids between a genetically engineered (GE) rice line (EP3) and two wild rice populations (w1 or w2) after the 25-day soil-burial treatment.

Hybrid Seed	F_4_		F_5_		F_6_	
	GE	Non-GE	GE	Non-GE	GE	Non-GE
EP3–w1	27.9 ± 2.2 ^1^	17.9 ± 1.2 ** ^2^	25.4 ± 1.4	18.3 ± 2.2 *	27.5 ± 2.4	19.2 ± 1.8 *
EP3–w2	25.4 ± 2.1	16.7 ± 1.2 **	23.8 ± 1.1	18.3 ± 2.0 *	28.3 ± 0.5	18.8 ± 1.9 **

^1^ Numbers following the averages indicate the standard error (*n* = 6 replicates).^2^ Comparisons were made between the GE and non-GE seeds based on the independent-sample Student’s *t*-test. * *p* < 0.05; ** *p* < 0.01.

**Table 2:** Average contents (ng/g) of five plant hormones in genetically engineered (GE) and non-GE F_4_–F_6_ dry seeds from hybrid lineages derived from crosses between a GE rice line (EP3) and two wild rice populations (w1 or w2).

Hybrid Lineage	Hormone	F_4_		F_5_		F_6_	
		GE	Non-GE	GE	Non-GE	GE	Non-GE
EP3–w1	ABA	90.3 ± 4.7 ^1^	65.5 ± 7.2 * ^2^	76.4 ± 5.8	65.3 ± 1.4 ^NS^	84.0 ± 5.9	82.3 ± 9.0 ^NS^
	*cis*-OPDA	3.7 ± 1.4	3.1 ± 1.3 ^NS^	4.1 ± 0.8	3.0 ± 0.3 ^NS^	4.0 ± 0.2	4.5 ± 0.4 ^NS^
	GA_3_	1.2 ± 0.1	1.1 ± 0.2 ^NS^	1.1 ± 0.2	1.1 ± 0.2 ^NS^	1.2 ± 0.2	1.1 ± 0.2 ^NS^
	JA	4.6 ± 0.4	7.4 ± 0.4 **	7.4 ± 0.4	6.7 ± 0.9 ^NS^	7.0 ± 1.1	6.9 ± 0.6 ^NS^
	SA	921.0 ± 102.1	816.8 ± 98.6 ^NS^	817.5 ± 55.4	822.2 ± 42.7 ^NS^	910.8 ± 73.9	957.0 ± 12.1 ^NS^
EP3–w2	ABA	75.2 ± 5.7	62.8 ± 5.3 ^NS^	88.9 ± 3.2	63.3 ± 7.9 ^*^	75.7 ± 7.2	64.0 ± 5.8 ^NS^
	*cis*-OPDA	3.5 ± 0.3	3.5 ± 0.5 ^NS^	4.0 ± 0.1	4.0 ± 0.0 ^NS^	4.0 ± 0.0	4.6 ± 0.4 ^NS^
	GA_3_	1.4 ± 0.4	1.1 ± 0.3 ^NS^	1.0 ± 0.3	1.2 ± 0.2 ^NS^	1.0 ± 0.1	1.1 ± 0.1 ^NS^
	JA	7.5 ± 0.5	6.6 ± 1.0 ^NS^	5.7 ± 1.4	4.8 ± 0.7 ^NS^	6.2 ± 0.1	6.7 ± 0.4 ^NS^
	SA	847.4 ± 26.9	856.2 ± 19.3 ^NS^	876.5 ± 18.0	959.0 ± 46.3 ^NS^	905.8 ± 73.9	887.5 ± 48.2 ^NS^

^1^ Numbers following the averages indicate the standard error (*n* = 3 replicates). ^2^ Comparisons were made between the GE and non-GE seeds based on the independent-sample Student’s *t*-test. ^NS^ no significance; * *p* < 0.05; ** *p* < 0.01.

**Table 3:** Average contents (ng/g) of five plant hormones in genetically engineered (GE) and non-GE F_4_–F_6_ imbibed seeds from hybrid lineages derived from crosses between a GE rice line (EP3) and two wild rice populations (w1 or w2).

Hybrid Lineage	Hormone	F_4_		F_5_		F_6_	
		GE	Non-GE	GE	Non-GE	GE	Non-GE
EP3–w1	ABA	53.8 ± 5.0 ^1^	43.0 ± 3.9 ^NS 2^	42.5 ± 8.3	35.4 ± 3.7 ^NS^	60.0 ± 2.7	46.4 ± 9.0 ^NS^
	*cis*-OPDA	1.3 ± 0.1	1.4 ± 0.1 ^NS^	1.3 ± 0.3	1.6 ± 0.7 ^NS^	1.8 ± 0.5	2.7 ± 0.2 ^NS^
	GA_3_	4.4 ± 0.6	3.4 ± 0.0 ^NS^	3.2 ± 0.1	3.3 ± 0.3 ^NS^	4.5 ± 1.1	4.0 ± 0.3 ^NS^
	JA	2.5 ± 0.7	1.8 ± 0.1 ^NS^	1.4 ± 0.2	1.5 ± 0.1 ^NS^	2.3 ± 0.2	1.7 ± 0.3 ^NS^
	SA	660.6 ± 127.5	493.4 ± 82.4 ^NS^	669.5 ± 50.6	732.8 ± 7.5 ^NS^	566.6 ± 77.4	717.8 ± 35.9 ^NS^
EP3–w2	ABA	55.3 ± 6.6	42.9 ± 2.5 ^NS^	29.4 ± 2.3	32.6 ± 4.6 ^NS^	46.2 ± 7.8	37.1 ± 2.8 ^NS^
	*cis*-OPDA	1.2 ± 0.1	1.3 ± 0.4 ^NS^	1.6 ± 0.3	1.0 ± 0.3 ^NS^	2.0 ± 0.2	2.3 ± 0.4 ^NS^
	GA_3_	3.7 ± 0.1	3.8 ± 0.4 ^NS^	4.8 ± 3.8	3.8 ± 0.4 ^NS^	4.5 ± 0.9	5.4 ± 0.1 ^NS^
	JA	2.7 ± 1.0	1.5 ± 0.4 ^NS^	1.2 ± 0.1	1.0 ± 0.1 ^NS^	2.0 ± 0.6	1.3 ± 0.1 ^NS^
	SA	722.7 ± 35.1	636.5 ± 33.6 ^NS^	680.6 ± 35.9	581.2 ± 124.9 ^NS^	605.2 ± 51.9	697.5 ± 64.6 ^NS^

^1^ Numbers following the averages indicate the standard error (*n* = 3 replicates). ^2^ Comparisons were made between the GE and non-GE seeds based on the independent-sample Student’s *t*-test. ^NS^ no significance.

## Data Availability

All the data was shown in the article.
